# High-Throughput Docking and Molecular Dynamics Simulations towards the Identification of Potential Inhibitors against Human Coagulation Factor XIIa

**DOI:** 10.1155/2020/2852051

**Published:** 2020-05-22

**Authors:** Dongfang Xu, Guangpu Xue, Bangya Peng, Zanjie Feng, Hongling Lu, Lihu Gong

**Affiliations:** ^1^Library of Zunyi Medical University, China; ^2^Institute of Chemistry and Biochemistry, Freie Universität Berlin, Germany; ^3^Department of Biochemistry, Zunyi Medical University, China

## Abstract

Human coagulation factor XIIa (FXIIa) is a trypsin-like serine protease that is involved in pathologic thrombosis. As a potential target for designing safe anticoagulants, FXIIa has received a great deal of interest in recent years. In the present study, we employed virtual high-throughput screening of 500,064 compounds within Enamine database to acquire the most potential inhibitors of FXIIa. Subsequently, 18 compounds with significant binding energy (from -65.195 to -15.726 kcal/mol) were selected, and their ADMET properties were predicted to select representative inhibitors. Three compounds (Z1225120358, Z432246974, and Z146790068) exhibited excellent binding affinity and druggability. MD simulation for FXIIa-ligand complexes was carried out to reveal the stability and inhibition mechanism of these three compounds. Through the inhibition of activated factor XIIa assay, we tested the activity of five compounds Z1225120358, Z432246974, Z45287215, Z30974175, and Z146790068, with pIC50 values of 9.3∗10^−7^, 3.0∗10^−5^, 7.8∗10^−7^, 8.7∗10^−7^, and 1.3∗10^−6^ M, respectively; the AMDET properties of Z45287215 and Z30974175 show not well but have better inhibition activity. We also found that compounds Z1225120358, Z45287215, Z30974175, and Z146790068 could be more inhibition of FXIIa than Z432246974. Collectively, compounds Z1225120358, Z45287215, Z30974175, and Z146790068 were anticipated to be promising drug candidates for inhibition of FXIIa.

## 1. Introduction

Human coagulation factor XIIa (FXIIa) is an important component involved in the initiation of the intrinsic pathway of the coagulation cascade [1]. The intrinsic coagulation cascade is initiated by contact activation in reactions involving high-molecular-weight kininogen (HMWK) and prekallikrein (PPK) [2]. The contact system can be activated by diverse negatively charged polymers, including kaolin, nucleic acids [3], and collagen [4]. Contacting with polyanions, FXII undergoes a conformational change and converts into active form *α*-FXIIa [5]. Essentially, kallikrein cleaves the FXII Arg353–Val354 peptide bond, generating *α*-FXIIa, which consists of a N-terminal heavy chain with a molecular weight of 50 kDa and a Cys340–Cys467 disulfide bridge-connected C-terminal light chain with a molecular weight of 28 kDa. The N-terminal heavy chain comprises a contact-binding domain, while the C-terminal light chain comprises a catalytic center. Once a small amount of *α*-FXIIa generate, it can further cleave prekallikrein to generate kallikrein. *α*-FXIIa and kallikrein then form a synergetic system to amplify their production. *α*-FXIIa activates factor XI to factor XIa, which contributes to plasma coagulation [6]. Subsequent cleavage of *α*-FXIIa results in loss of the heavy chain and generation of the isolated protease domain termed *β*-FXIIa, consisting of a nine amino acid peptide heavy chain remnant disulfide bonded to the protease domain [5, 7–9].

Inhibition of FXII activity is an attractive approach for the treatment and prevention of thrombotic diseases [10]. Previous studies show that FXII-deficient mice are protected from arterial thrombosis, ischemic stroke, and deep vein thrombosis while maintaining normal hemostasis [11, 12], suggesting that FXII is critically involved in pathologic thrombus formation but is dispensable for hemostasis. For more than five decades, it has been known that deficiency of coagulation factor XII is not associated with increased spontaneous or injury-related bleeding complications [13]. Patients that are deficient in FXII do not suffer from abnormal bleeding even during major surgical procedures [14]. This observation and the limited role of FXII in hemostasis raised the prospect that inhibiting FXIIa could offer an antithrombotic therapy with a low bleeding risk [15].

In recent years, inhibitors for FXIIa have been generated and described in various preclinical models in vitro or in vivo [16]. These agents include monoclonal antibodies [13, 17], natural peptide or protein inhibitors [18, 19], small molecule inhibitors [12, 20], RNA aptamer [21], small interfering RNA [22], and antisense oligonucleotide [23]. Currently available antithrombotic agents such as heparin, warfarin, and antiplatelet therapy can cause serious bleeding complication, because they target components of the blood-clotting mechanism such as thrombin, FVIIa, FIXa, FXa, and FXIa [24]. Therefore, it is necessary to design new FXIIa inhibitors and optimize them into therapeutic agents, which have high inhibitory activity against FXIIa without increasing the bleeding risk and exist stably in human plasma.

Structure-based drug design relies on the knowledge of the three-dimensional structure of the biomolecular target. It is extensively applied to find potent and specific inhibitors against a particular drug [25–27]. This study reports the identification and validation of novel FXIIa inhibitors using high-throughput screening (HTS), docking, and molecular dynamics (MD) simulation-based approaches. Finally, MD simulations were performed on FXIIa, FXIIa-Z1225120358 complex, FXIIa-Z432246974 complex, and FXIIa-Z146790068 complex to get deeper insights into the binding mechanism of FXIIa to the selected ligands.

## 2. Materials and Methods

### 2.1. Materials

The study was carried out on HP Workstation with 3.5 GHz processor, 8 GB RAM, and 1 TB hard drive running in Windows operating system, high speed internet (broadband) connection, and uninterrupted and stabilized power supply. Bioinformatics software: Discovery Studio, GraphPad Prism software, and online resources, like NCBI, RCSB, and Enamine database, were used in this study.

Full length-activated FXIIa (a-FXIIa) were obtained from Enzyme Research Laboratories (Swansea, UK). Commercial compounds were obtained from Enamine (KIEV, Ukraine). S2302 (a chromogenic substrate peptide mimic) was obtained from Chromogenix (Epsom, UK).

### 2.2. Databases Used for Screening

Enamine Library, a free database of commercially available compounds, was used to perform virtual screening. This approach allowed the identification of 500,064 compounds, which contains kinase inhibitor library.

### 2.3. High-Throughput Screening

The docking studies were based on the available crystal structure of FXIIa (PDB code: 6B77). Water molecules and heteroatom including ligands were removed. Compounds obtained from the Enamine database were docked into the FXIIa protein active site. HTS was then performed using the Libdock method, which has been incorporated into Discovery Studio 2018 (Discovery Studio User Manual).

Before docking, the receptor structure was typed with CHARMM force field [28], hydrogen atoms were added, and pH of the protein was adjusted to almost neutral 7.4 using protein preparation module. All ligands were typed with CHARMM force field and minimized by using the smart minimizer minimization algorithm of Discovery Studio 2018, which contains 1000 steps of steepest descent with a RMS gradient tolerance of 3 and conjugate gradient minimization. The ten top-scoring conformations of every ligand were then saved at the end of calculation. For all compounds, the docked structures with the highest scores were analyzed to identify the molecules. Finally, the complexes of 18 top-ranking compounds with 6B77 were subjected to free energy calculations.

### 2.4. Binding Free Energy Calculation

To estimate the binding energy between a receptor and a ligand, we calculated the average binding energy across a set of related poses [29]. The binding energy is calculated using the following equation: Energy_Binding_ = Energy_Complex_ − Energy_Ligand_ − Energy_Receptor_. The Binding Free Energy Calculation was carried out using the Discovery Studio 2018 program.

### 2.5. ADMET Prediction

Binding affinities based on binding energy, top 18 hits were selected which showed a significantly higher binding affinity towards the binding pocket of FXIIa. All these compounds were further subjected to ADMET prediction to get drug-like molecules. As a successful small molecular drug, it should not only be active against a target but also possess appropriate ADMET properties. ADMET properties play vital roles in the discovery and development of small molecule drugs [30]. We have further performed ADMET Descriptors and Toxicity Prediction methods in Discovery Studio 2018. These ADMET modules include aqueous solubility, BBB penetration, cytochrome P450 (CYP450) 2D6 inhibition, hepatotoxicity, human intestinal absorption (HIA), and plasma protein binding. The data for establishing these modules are derived from a large number of literature reports and experimental data, and these models have been widely validated (Discovery Studio User Manual).

### 2.6. Molecular Dynamics Simulations

MD simulations were performed on FXIIa, FXIIa-Z1225120358 complex, FXIIa-Z432246974 complex, and FXIIa-Z146790068 complex. All simulations were carried out with Discovery Studio 2018. The CHARMM force field was applied to both protein and small molecules. The protein-ligand systems were solvated for the simulation, adding enough water molecules to allow the protein to interact with the solvent naturally. The protein should be solvated in a water box, which allows the simulation to run using periodic boundary conditions to avoid surface artifacts [31].

Initially, the system underwent 1000 steps of steepest descent minimization and 2000 steps of conjugate gradient minimization. The energy minimization step was followed by heating, equilibration, and production. The whole system was heated from an initial temperature of 50 K to 300 K in 4 ps without restraint. The equilibration was run in 300 K for 20 ps without restraint. The production was run in 300 K for a time of 200 ps with typed NPT. The total simulation time over the heating, equilibration, and production steps is 224 ps. The electrostatic parameter was set to automatic, which automatically recognizes the periodic environment and sets the electrostatic calculation to Particle Mesh Ewald (PME). RMSD and RMSF were computed for the entire protein molecule using the starting structure as reference. Hydrogen bonding between the FXIIa protein and the compound was monitored and analyzed over the course of the simulation [32].

### 2.7. FXIIa Enzymatic Assay

The enzymatic activity of a-FXIIa was measured by monitoring the amount of pNA chromophore released from substrate H-D-Pro-Phe-Arg-pNA (S-2302). Assays were performed in a 100 *μ*L volume at 33°C in 96-well plates in a PerkinElmer (Seer Green, UK) Envision plate reader, and pNA release was followed over a period of 6 h by reading the absorbance at 405 nm. Absorbance values were converted to pNA concentrations by comparison with a standard curve obtained under exactly the same instrument conditions. All absorbance values were within the linear measurement range of the instrument. Initial rates were calculated on the basis of the first 30 min of incubation. To assay inhibitors, concentrations of five compounds (Z1225120358, Z45287215, Z30974175, Z432246974, and Z146790068) between 10^−8^ M and 10^−3^ M were incubated with 200 *μ*M substrate peptide, the reaction was initiated by addition of 10 nM a-FXIIa, enzymatic activity was measured as described above, and IC 50 values were determined by nonlinear regression analysis (GraphPad Prism, version 8.0.2.263) using the log[inhibitor] versus response variable slope algorithm with a bottom constraint.

## 3. Result and Discussion

HTS followed by docking and MD simulation were used to find the potential inhibitors of human FXIIa. A total of 500,064 compounds were screened with the structure of FXIIa, and top-ranked 18 molecules with binding affinity to FXIIa (ΔG ≤ −10 kcal/mol) were selected for further analysis (Table 1). All novel hits were accurately fitted within the active site of FXIIa and were further evaluated with druggability parameters.

### 3.1. High-Throughput Screening and Binding Free Energy Calculation

500,064 compounds from Enamine databases were virtually screened onto the structure of FXIIa. The structures of the 18 compounds were successfully docked with a binding energy range from -65.195 kcal/mol to -15.726 kcal/mol [33]. Compound Z45287215 showed the highest binding affinity of -65.195 kcal/mol. The result of this screening is listed in Table 1, and the property contained hydrogen bond donors, hydrogen bond acceptors, binding energy, AlogP, and molecular weight (MW).

### 3.2. Analysis of the ADMET Prediction

ADMET properties have become one of the most key issues to assess the efficacies or risks of small molecular compounds in biology [34]. The AMDET properties of small molecular compounds could be predicted by integrally analyzing their physicochemical properties, such as molecular weight (MW), polar surface area (PSA), lipophilicity (clogP), and aqueous solubility (logS), which are directly correlated with properties of drug molecules like absorption and bioavailability [35]. We applied the Discovery Studio ADMET tool to predict the pharmacokinetic profiles of compounds (Table 2).

The blood brain barrier penetration ability of a compound is another equally important parameter for a drug molecule [36]. We found that almost all compounds in Enamine Library are high penetrative except compounds Z30974175, Z432246974, Z818810338, and Z914249910. Human intestinal absorption is given by four prediction levels: “0” (good), “1” (moderate), “2” (poor), and “3” (very poor). The absorption levels of all the compounds are all “0,” showing a satisfactory level of absorption. The classification whether a compound is highly bound (≥90% bound) to plasma proteins using the cutoff Bayesian score of -2.209, the results indicate that compounds Z1225120358, Z432246974, Z56867305, Z818810338, Z146790068, and Z1392999050 show strong binding affinity. Almost all compounds are unable to inhibit the CYP450 enzyme activity except compound Z45287215.

All compounds in this study are noncarcinogenic and nonmutagenic except Z30974175, Z53058673, Z56867305, Z223449194, Z818810338, Z19630209, Z603981096, Z53058577, Z132701382, Z603981096, and Z1392999050. Calculated ADME values and other structural properties of the FXIIa bound ligands including carcinogenicity mutagenicity are listed in Table 2.

### 3.3. Structure Analysis

The FXIIa specificity pocket bordered by segments Ile213-Cys220, Asp189-Ser195, Pro225-Thr229, and disulfide bond Cys191-Cys220 is practically identical to that of other active trypsin-like serine proteases. According to the ADMET properties of all compounds, we found that compounds Z1225120358, Z432246974, and Z146790068 have the highest binding affinity towards FXIIa. The AMDET properties of Z45287215 show not well but have the highest binding affinity of -65.195 kcal/mol.

**Figure 1:**
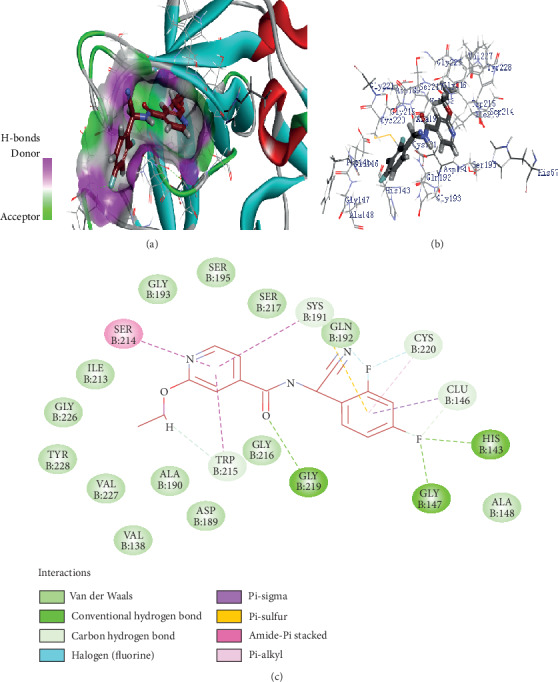
Binding mode of Z1225120358 to the FXIIa. (a) Overall structure of FXIIa-Z1225120358 complex. Protein structure was shown in cartoon and ligand in sticks. Showing the binding site residue surface around the ligand. Displaying the H-bond donor (purple) and acceptor (green) area. (b) Interactions between Z1225120358 (sticks) and FXIIa residues (sticks). (c) 2D diagram of FXIIa interactions to compound Z1225120358.

Structure analysis shows that Z1225120358 binds to the active site cavity of the FXIIa. The estimated binding affinity is significantly high (-39.884 kcal/M) (Figure 1(a)). Three hydrogen bond interactions were observed (His143, Gly147, and Gly219) for Z1225120358 to FXIIa, as well as many van der Waals interactions offered by Asp189, Ala190, Gln192, Ile213, Gly216, Ser217, Gly226, Val227, and Tyr228 (Figures 1(b) and 1(c)). The complex of FXIIa-Z1225120358 is stabilized by forming noncovalent interaction between Z1225120358 and FXIIa residues.

**Figure 2:**
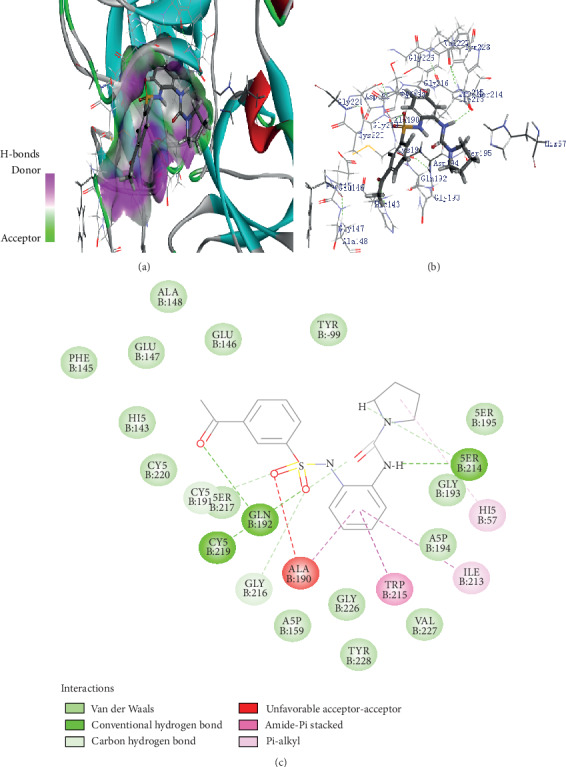
Binding mode of Z432246974 to the FXIIa. (a) Overall structure of FXIIa-Z432246974 complex showing protein in cartoon model and ligand in stick. Showing the binding site residue surface around the ligand. Displaying the H-bond donor (purple) and acceptor (green) area. (b) Interactions of Z432246974 (sticks) to the FXIIa residues (sticks). (c) 2D diagram of FXIIa interactions to compound Z432246974.

In another complex, Z432246974 binds to the active site cavity of the FXIIa. The estimated binding affinity is also significantly high (-36.396 kcal/M) (Figure 2(a)). There are three hydrogen bond interactions observed (Gln192, Ser214, and Gly219) for Z432246974 to the FXIIa and many van der Waals interactions offered by Tyr99, Ala190, Glu146, Ile213, Gly147, Asp189, Cys220, Ser217, Gly226, Val227, and Tyr228 (Figures 1(b) and 1(c)). The complex of FXIIa-Z1225120358 is stabilized by forming several noncovalent interactions with FXIIa residues.

**Figure 3:**
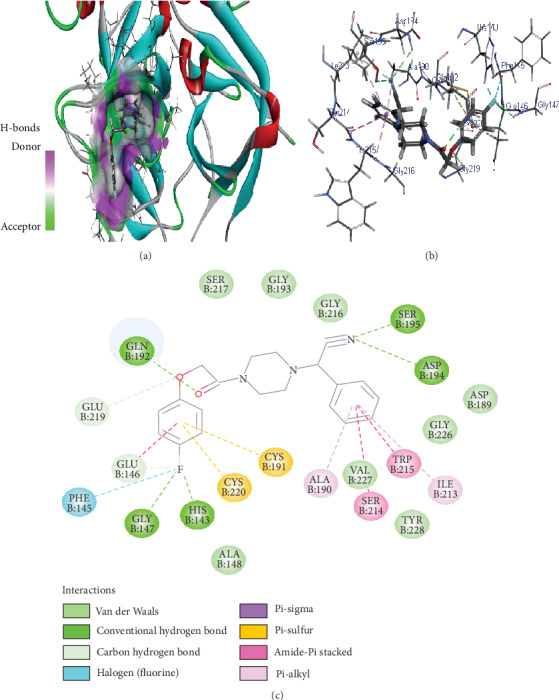
Binding mode of Z432246974 to the FXIIa. (a) Overall structure of FXIIa-Z146790068 complex showing protein in cartoon model and ligand in stick. Showing the binding site residue surface around the ligand. Displaying the H-bond donor (purple) and acceptor (green) area. (b) Interaction of Z146790068 (stick) to the FXIIa residues (stick). (c) 2D diagram of FXIIa interaction to compound Z146790068.

Similarly, Z146790068 also binds to the active site cavity of FXIIa with a binding affinity of -25.434 kcal/M (Figure 3(a)). There are five hydrogen bond interactions observed for Z146790068 to the FXIIa residues (His143, Gly147, Gln192, Asp194, and Ser195) and many van der Waals interactions offered by Phe145, Glu146, Ala190, Cys191, Ile213, Ser214, Trp215, Gly216, and Gly219 (Figures 3(b) and 3(c)). The complex of FXIIa-Z146790068 is stabilized by the formation of several noncovalent interactions offered by FXIIa residues.

**Figure 4:**
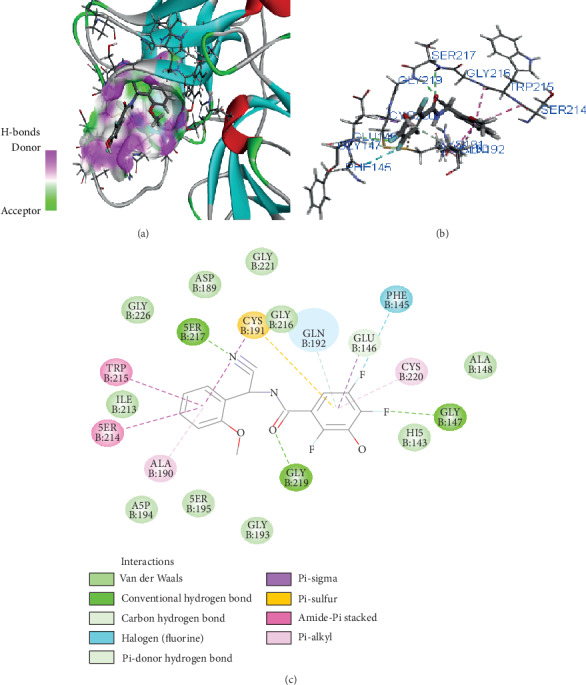
Binding mode of Z45287215 to the FXIIa. (a) Overall structure of FXIIa-Z45287215 complex showing protein in cartoon model and ligand in stick. Showing the binding site residue surface around the ligand. Displaying the H-bond donor (purple) and acceptor (green) area. (b) Interaction of Z45287215 (stick) to the FXIIa residues (stick). (c) 2D diagram of FXIIa interaction to compound Z45287215.

Compound Z45287215 showed the highest binding affinity of -65.195 kcal/mol, Three hydrogen bond interactions were observed (Gly147, Ser217, and Gly219) for Z45287215 to FXIIa, as well as many van der Waals interactions offered by Asp189, Ala190, Gln192, Ile213, Gly216, Ser217, Gly226, Val227, and Tyr228 (Figures 4(b) and 4(c)). The complex of FXIIa-Z45287215 is stabilized by forming noncovalent interaction between Z45287215 and FXIIa residues.

### 3.4. Molecular Dynamics Simulation

To further analyze protein-ligand interactions, we selected the top three *ADMET* compounds for molecular dynamics simulation. MD simulations were performed for FXIIa, FXIIa-Z1225120358 complex, FXIIa-Z432246974 complex, and FXIIa-Z146790068 complex by 200 ps. The constant temperature fluctuations at 300 K for each system suggest a stable and accurate nature of the MD simulations. The average potential energy of FXIIa, FXIIa-Z1225120358 complex, FXIIa-Z432246974 complex, and FXIIa-Z146790068 complex was analyzed. An average potential energy for FXIIa, FXIIa-Z1225120358 complex, FXIIa-Z432246974 complex, and FXIIa-Z146790068 complex was found to be -75682 kJ/mol, -76531 kJ/mol, -76632 kJ/mol, and -76594 kJ/mol, respectively.

**Figure 5:**
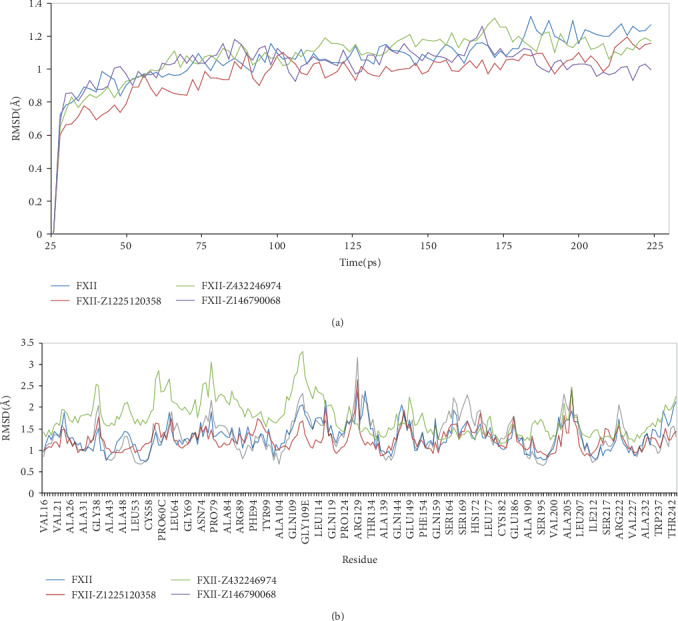
RMSD values (a) and RMSF values (b) of FXIIa and its complexes with inhibitors as a function of time obtained for MD simulation.

The RMSD value is used to measure the structural alterations in MD simulation [37]. As seen in Figure 5(a), the RMSD values of each simulation-maintained fluctuations are all less than 1.1 Å. The average root mean square deviation (RMSD) values were found to be 1.064 Å, 0.963 Å, 1.076 Å, and 1.025 Å for FXIIa, FXIIa-Z1225120358 complex, FXIIa-Z432246974 complex, and FXIIa-Z146790068 complex, respectively. The binding of Z1225120358 and Z146790068 to the FXIIa leads to a decrease in the RMSD values, whereas the binding of Z432246974 to the FXIIa leads to an increase of RMSD value. The results indicate that Z1225120358 and Z146790068 compounds tightly bind to the active pocket of FXIIa, whereas bound by compound Z432246974 leads to higher structural deviations of FXIIa. On this basis, fluctuations of FXIIa residues were declined and the activity of FXIIa may be inhibited by compounds Z1225120358 and Z146790068.

RMSF value is considered as the criterion of the overall flexibility in MD simulation [38, 39]. Moreover, we also investigated the motion of key residues of FXIIa interacted with inhibitors [35]. As shown in Figure 5(b), the RMSF values of FXIIa-Z1225120358 complex and FXIIa-Z146790068 complex are less than that of FXIIa, while the RMSF value for FXIIa-Z432246974 complex is greater.

**Figure 6:**
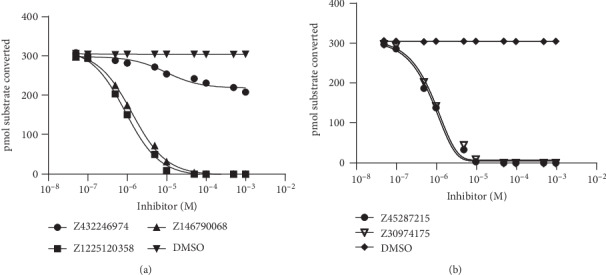
Inhibition of activated factor XII (a-FXIIa) by five compounds Z1225120358, Z45287215, Z30974175, Z432246974, and Z146790068. Concentrations of these five compounds (10^−8^ M to 10^−3^ M) were incubated with 200 *μ*M substrate peptide, and this was followed by addition of a-FXIIa; enzymatic activity was then monitored as described in Materials and Methods. pIC50 values were obtained by nonlinear regression (GraphPad Prism V8.0.2.263; log[inhibitor] versus response-variable slope algorithm with a bottom constraint). Error bars indicate the standard error (*n* = 3 independent observations).

Inhibition of activated factor XII (a-FXIIa) by five compounds Z1225120358, Z432246974, Z45287215, Z30974175, and Z146790068, with pIC50 values of 9.3∗10^−7^, 3.0∗10^−5^, 7.8∗10^−7^, 8.7∗10^−7^, and 1.3∗10^−6^ M, respectively (Figure 6, Table 3). The results showing that compounds Z1225120358, Z45287215, Z30974175, and Z146790068 could be more inhibition of FXIIa than Z432246974. The AMDET properties of Z45287215 and Z30974175 show not well but have better inhibition activity. The results of biological activity experiments combined with structural analysis. Three hydrogen bond interactions were observed (His143, Gly147, and Gly219) for Z1225120358 to FXIIa, and three hydrogen bond interactions were observed (Gly147, Ser217, and Gly219) for Z45287215 to FXIIa. His143, Ser217, Gly147, and Gly219 in the FXIIa specificity pocket play an important role in inhibiting FXII activity.

## 4. Conclusions

FXII is an emerging promising target for serious diseases. It plays important roles in thrombosis, hemostasis, and additional pathologic settings [40]. We have identified three potential inhibitors of FXIIa using HTS and MD simulation. All these three compounds bind to the common residues of the active site cavity of the FXIIa. High-throughput virtual screening was performed by a docking method. The top eighteen candidate inhibitors were further selected by AMDET prediction. Following MD simulation for FXIIa-ligand complexes effectively revealed that all of candidate compounds bind to the FXIIa active site. MD simulation results showed that bond with compound Z432246974 leads to higher structural deviations for FXIIa, suggesting that compound Z432246974 is not stable. We also found that compound Z1225120358 and Z146790068 could be more inhibition of FXIIa than Z432246974. Inhibition of activated factor XII assay and MD simulation, which had the same result, confirm that the analytical theory of MD simulation was correct. Inhibition of activated factor XII (a-FXIIa) by five compounds Z1225120358, Z432246974, Z45287215, Z30974175, and Z146790068, with pIC50 values of 9.3∗10^−7^, 3.0∗10^−5^, 7.8∗10^−7^, 8.7∗10^−7^, and 1.3∗10^−6^ M, respectively. The AMDET properties of Z45287215 and Z30974175 show not well but have better activity than Z1225120358. The results of biological activity experiments combined with structural analysis, His143, Ser217, Gly147, and Gly219 in the FXIIa specificity pocket, which play an important role in inhibiting FXII activity. In conclusion, compounds Z1225120358, Z45287215, Z30974175, and Z146790068 were anticipated to be promising drug candidates for inhibition of FXIIa. Further experimental validations are required to confirm the inhibitory potential of these ligands against FXIIa.

## Figures and Tables

**Table 1:** Compounds selected as potential FXIIa inhibitors by HTS filter and Binding Free Energy Calculations.

No.	Compound id	MW	Binding energy (kcal/mol)	AlogP	H-bond donor	H-bond acceptor	Chemical structure
1	Z45287215	310.372	-65.1947	3.4685	1	1	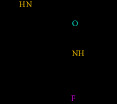
2	Z30974175	424.303	-50.0193	3.6279	2	3	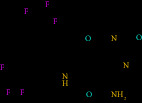
3	Z1225120358	317.296	-39.8838	2.6222	1	4	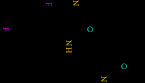
4	Z53058673	330.387	-39.6591	3.0745	1	2	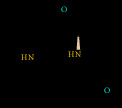
5	Z53059201	341.414	-38.4803	2.9696	1	2	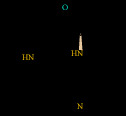
6	Z432246974	387.45	-36.396	2.8377	2	4	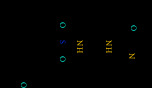
7	Z56867305	300.314	-34.1774	3.2588	1	2	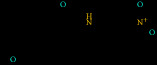
8	Z223449194	313.33	-30.3052	3.3693	1	2	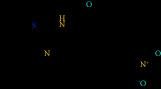
9	Z818810338	430.464	-26.6265	3.736	1	4	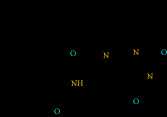
10	Z146790068	353.397	-25.4344	2.6916	0	3	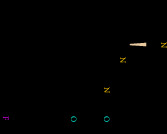
11	Z53059185	341.414	-23.701	3.1847	1	2	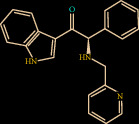
12	Z19630209	393.399	-22.6212	1.5979	1	4	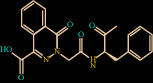
13	Z603981096	348.406	-20.2983	3.8445	2	3	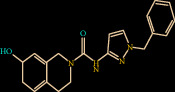
14	Z53058577	344.414	-19.9549	3.3227	1	2	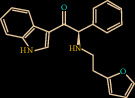
15	Z132701382	344.414	-17.8038	3.5587	1	2	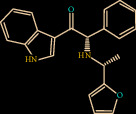
16	Z603981096	348.406	-17.7091	3.8445	2	3	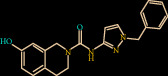
17	Z914249910	322.412	-17.1128	2.6614	1	3	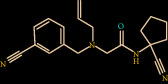
18	Z1392999050	360.361	-15.7256	3.2774	1	4	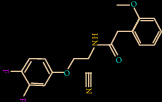

**Table 2:** The ADMET prediction results of inhibitors.

No.	Compound id	Solubility	Absorption level	CYP2D6 inhibitor	BBB	BBB LEVEL	PPB	PPB	PSA	ogP98	Ames mutagenicity	Carcinogenicity
1	Z45287215	-5.288	0	True	0.363	1	-3.123	False	45.166	3.984	Nonmutagen	Non
2	Z30974175	-5.754	0	False	-0.899	3	-3.97672	False	97.957	2.604	Mutagen	Yes
3	Z1225120358	-3.705	0	False	-0.503	2	0.170651	True	73.237	2.619	Nonmutagen	Non
4	Z53058673	-5.208	0	False	0.113	1	-8.74339	False	57.72	3.817	Nonmutagen	Yes
5	Z53059201	-4.92	0	False	0.03	1	-11.4012	False	56.427	3.485	Nonmutagen	Non
6	Z432246974	-3.282	0	False	-1.139	3	4.41143	True	98.176	1.84	Nonmutagen	Non
7	Z56867305	-4.232	0	False	-0.442	2	3.37623	True	81.864	3.258	Mutagen	Yes
8	Z223449194	-5.198	0	False	-0.329	2	5.26529	True	84.195	3.744	Mutagen	Non
9	Z818810338	-4.591	0	False	-0.634	3	-0.75978	True	94.066	3.264	Nonmutagen	Yes
10	Z146790068	-3.545	0	False	-0.206	2	1.81081	True	55.87	2.691	Nonmutagen	Non
11	Z53059185	-5.107	0	False	0.097	1	-9.41512	False	56.427	3.7	Nonmutagen	Non
12	Z19630209	-2.668	0	False	N/A	4	-5.10215	False	117.504	1.497	Nonmutagen	Yes
13	Z603981096	-4.033	0	False	-0.243	2	-4.36726	False	70.888	3.34	Nonmutagen	Yes
14	Z53058577	-5.313	0	False	0.189	1	-8.47245	False	57.72	4.066	Nonmutagen	Yes
15	Z132701382	-5.703	0	False	0.262	1	-8.8002	False	57.72	4.302	Nonmutagen	Yes
16	Z603981096	-4.033	0	False	-0.243	2	-4.36726	False	70.888	3.34	Nonmutagen	Yes
17	Z914249910	-3.283	0	False	-0.587	3	-2.42367	False	79.333	2.661	Nonmutagen	Non
18	Z1392999050	-4.152	0	False	-0.263	2	4.96262	True	70.906	3.277	Nonmutagen	Yes

**Table 3:** Inhibition of activated factor XII (a-FXIIa) by five compounds.

Compounds	Z1225120358	Z432246974	Z45287215	Z30974175	Z146790068
pIC50 values (M)	9.3∗10^−7^	3.0∗10^−5^	7.8∗10^−7^	8.7∗10^−7^	1.3∗10^−6^

## Data Availability

The underlying data in this manuscript were taken from the study by Zunyi Medical University and are cited in this study.

## Abbreviations

FXIIa: Human blood coagulation factor XIIa
MD: Molecular dynamics
HTS: High-throughput screening
ADMET: Absorption, distribution, metabolism, excretion, toxicity
RMSD: Root mean square deviation
RMSF: Root mean square fluctuation.
